# Effect of Occupational Stress on Cognitive Functions Among Resident Doctors

**DOI:** 10.7759/cureus.113425

**Published:** 2026-07-26

**Authors:** Mohammad S Raza, Farahanaz B Irani, Pranita M Kadam

**Affiliations:** 1 Physiology, Mahatma Gandhi Mission's (MGM) Medical College and Hospital, MGM Institute of Health Sciences, Aurangabad, IND

**Keywords:** attention, cognitive function, executive function, occupational stress, postgraduate residents, working memory

## Abstract

Background

Occupational stress is highly prevalent among postgraduate medical residents due to heavy workload, prolonged duty hours, emergency responsibilities, and sleep deprivation. Chronic stress may adversely affect cognitive functions, compromising both the physician's well-being and patient care. This study aimed to assess occupational stress among postgraduate resident doctors and evaluate its effect on cognitive functions.

Methods

A cross-sectional observational study was conducted in the Department of Physiology, Mahatma Gandhi Mission Medical College and Hospital, Aurangabad, Maharashtra, India, from April 2024 to March 2026. A total of 281 resident doctors aged 25-45 years were enrolled and categorized into clinical, para-clinical, and pre-clinical departments. Occupational stress was assessed using the Professional Psychological Stress Test based on the David Fontana protocol. Cognitive functions, including attention, perception, execution, and working memory, were evaluated using standardized computerized cognitive tests. Data were analyzed using Statistical Product and Service Solutions (SPSS, version 24; IBM SPSS Statistics for Windows, Armonk, NY). Quantitative variables were expressed as mean ± SD and categorical variables as proportions. Chi-square test, one-way ANOVA, unpaired t-test, and Pearson's correlation coefficient were used, with p < 0.05 considered statistically significant.

Results

A total of 281 resident doctors were enrolled, including 239 (85.1%) clinical, 31 (11.0%) para-clinical, and 11 (3.9%) pre-clinical residents. Moderate occupational stress was observed in 133 (47.3%) participants, while 126 (44.8%) experienced high stress, with significantly higher stress levels among clinical residents (p<0.00001). Clinical residents also had significantly longer working hours, more emergency duty calls, shorter sleep duration, and higher systolic and diastolic blood pressure than para-clinical and pre-clinical residents (all p<0.001). Cognitive assessment demonstrated significantly poorer attention, perception, and executive function among clinical residents (p<0.05), whereas working memory did not differ significantly across departments. In the clinical group, occupational stress showed significant positive correlations with impaired attention (r=0.172, p=0.008), executive function (r=0.249, p<0.0001), and working memory performance (r=0.340, p<0.0001), while no significant correlations were observed among para-clinical and pre-clinical residents.

Conclusion

Occupational stress is highly prevalent among postgraduate resident doctors, particularly in clinical specialties. Increased stress is associated with impaired cognitive functions, especially attention, executive function, and working memory. Heavy workload, sleep deprivation, unhealthy lifestyle habits, and elevated blood pressure further contribute to these adverse effects. Institutional stress-management strategies, promotion of healthy lifestyles, and mental health support are essential to improve resident well-being, cognitive performance, and quality of patient care.

## Introduction

Stress is a part of daily life, affecting everyone. Mild stress helps with work and motivation. Excess stress harms personality and health. Stress is the body's response to demands and may be helpful or harmful depending on management. Too much stress causes fatigue and illness [1].

Occupational stress is stress related to work. It includes mental and physical reactions to workplace pressure. Stress is the body's response to physical, emotional, or mental demands [2]. When demands exceed coping ability, stress becomes harmful and affects performance and well-being. Occupational stress develops from workload, conflicts, time pressure, and workplace expectations. Long-term stress can affect health, productivity, and job satisfaction. In the medical field, stress is very common among doctors, nurses, and postgraduate residents [3]. Burnout affects both residents' health and patient safety, as stressed doctors are more likely to make errors. Occupational stress also harms relationships, causing isolation and reduced social support [4].

Cognitive functions are mental processes that help people learn, think, and respond to the environment. They include attention, memory, decision-making, problem-solving, language, and visuospatial skills [5]. Cognitive functions are essential for daily activities and intellectual work. In medical training, they are important for clinical reasoning, diagnosis, and patient care. Attention helps monitor patients and notice changes. Memory helps recall medical knowledge and patient history. Executive functions support planning, decision-making, and emergency management. Language skills improve communication, while visuospatial skills help in imaging interpretation and surgical procedures [6].

Residency involves high cognitive demands. Residents must combine theory with practical skills under stress and time pressure. They manage many tasks at the same time, requiring strong attention and memory. Medical decisions often involve uncertainty, so quick thinking and cognitive flexibility are important [7]. Stress strongly affects cognitive functions. Acute stress may sometimes improve performance by increasing alertness and concentration, known as the "fight-or-flight" response [8]. However, chronic or excessive stress has harmful effects. Increased cortisol levels impair the functioning of the hippocampus, leading to difficulties in memory and learning. Stress also affects the prefrontal cortex, which is responsible for planning and decision-making [9].

Stress can greatly affect the cognitive functions of postgraduate medical residents. The present study aimed to assess the level of stress among residents, evaluate its impact on cognitive functions, and help in planning interventions to improve both physician well-being and clinical performance.

## Materials and methods

Study design and setting

This observational cross-sectional study was conducted in the Department of Physiology at Mahatma Gandhi Mission (MGM) Medical College and Hospital, Aurangabad, Maharashtra, India, from April 2024 to March 2026.

Ethical consideration

Ethical committee approval for the study was obtained from the Institutional Ethics Committee of MGM Medical College and Hospital (approval number: MGM-ECRHS/2024/76, dated March 30,2024).

Selected subjects were resident doctors working in MGM Medical College and Hospital, Aurangabad, and were willing to participate and provide informed consent, aged 25-45 years, of both genders. Exclusion criteria comprised of doctors with incomplete questionnaire responses, diagnosed with psychological disorders, on antipsychotic or antidepressant medication, or diagnosed with chronic illnesses, such as cancer or kidney disease. A total of 281 resident doctors meeting the selection criteria were recruited using convenience sampling after providing written informed consent and were included in the final analysis.

Data collection

Subjects were grouped into three based on postgraduate subjects (clinical, para-clinical, pre-clinical). After receiving written informed consent, individual interviews were conducted while ensuring privacy. Clinical history and findings of the clinical examination were recorded using a predesigned proforma.

The occupational stress score was assessed using the Professional Psychological Stress Test based on the David Fontana protocol [10]. The questionnaire included 25 multiple-choice questions. A score of 15 means stress is not a problem in your life; scores of 16-30 mean a moderate range of stress; and scores of 31-45 mean stress is clearly a problem.

Cognitive domains [11] were assessed using standardized online tests [12] and the following validated tasks:

Attention (go-no-go test): Attention was assessed using the go-no-go test. Participants were instructed to respond by clicking on a green dot (target stimulus) and to withhold their response when a patterned dot (non-target stimulus) appeared. The average reaction time (milliseconds) was recorded as a measure of attention and cognitive processing speed.

Perception (fast counting test): Perception was evaluated using the fast counting test, which assessed subitizing ability (rapid recognition of small quantities). Participants identified the number of dots (4-7) displayed briefly on the screen by pressing the corresponding number key. The average reaction time was calculated from 12 trials.

Executive function (colour reading interference test): Executive function was assessed using the colour reading interference test (modified Stroop test). Participants identified the colour of the displayed stimulus while ignoring the written word, requiring attentional shifting, selective attention, and cognitive control. Reaction times were recorded over 12 trials.

Working memory (two-back test): Working memory was assessed using the two-back test. Participants were required to identify whether the current stimulus matched the one presented two positions earlier in the sequence. The test consisted of 30 trials, and reaction time was recorded to evaluate working memory performance.

The cognitive function tests were based on reaction time, recorded in milliseconds. Longer reaction times indicated slower cognitive processing and were interpreted as poorer cognitive performance, suggesting a decline in the assessed cognitive function.

Data analysis

Data were entered and managed using Microsoft Excel (version Home 2024; Microsoft Corporation, Redmond, WA). Statistical analysis was performed using Statistical Product and Service Solutions (SPSS, version 24; IBM SPSS Statistics for Windows, Armonk, NY). Quantitative variables were expressed as mean ± SD, while categorical variables were expressed as proportions. A chi-square test was used to assess associations between variables. One-way ANOVA was used to compare stress and cognitive functions among departments. An unpaired t-test was used to compare variables. Pearson's correlation coefficient was used to assess the relationship between occupational stress score and cognitive function. P-value was checked at 5% level of significance.

## Results

This study enrolled 281 resident doctors to investigate the impact of occupational stress on their cognitive functions. Participants underwent a comprehensive assessment evaluating stress components, cognitive performance, and associated risk factors. The primary objective was to determine the effect of stress on cognitive abilities. Additionally, the study examined variations in stress levels and cognitive performance among resident doctors.

Table 1 shows that most participants (95.7%) were aged 25-30 years. There were 143 (50.9%) males and 138 (49.1%) females. Normal BMI was seen in 40.6%, overweight in 33.8%, and obesity in 25.6%. Mean BMI was 24.40 ± 2.36 kg/m². Participants included 239 clinical, 31 paraclinical, and 11 preclinical residents. Most participants (90.7%) were unmarried. A mixed diet was followed by 58.7%, while 41.3% were vegetarians. Irregular meal timing was reported by 85.8%. Smokers constituted 38.1%, while 29.9% consumed alcohol. Exercise was reported by 49.8% participants. Among them, 30.7% exercised regularly. Diabetes was present in 4.3% and hypertension in 8.9% of participants.

**Table 1:** Demographic profile of participants BMI: Body Mass Index, DM: Diabetes Mellitus, HTN: Hypertension

Parameters	Particulars	Frequency	Percentage
Age (in years)	25-30	269	95.7
	31-35	09	3.2
	>35	03	1.1
Gender	Male	143	50.9
	Female	138	49.1
BMI	Underweight (<18.5kg/m²)	00	00
	Normal (18.5-22.9 kg/m²)	114	40.6
	Overweight (23.0-24.9 kg/m²)	95	33.8
	Obese (≥25.0 kg/m²)	72	25.6
Departments	Clinical	239	85
	Para-clinical	31	11
	Pre-clinical	11	4
Marital Status	Married	26	9.3
	Unmarried	255	90.7
Diet Time	Veg	116	41.3
	Mixed	165	58.7
	Regular	40	14.2
	Irregular	241	85.8
Smoking	Yes	107	38.1
	No	174	61.9
Alcohol	Yes	84	29.9
	No	197	70.1
Exercise	Yes	140	49.8
	No	141	50.2
Regularity of Exercise [n=140]	Regular	43	30.7
	Irregular	97	69.3
History of HTN/DM	DM	12	4.3
	HTN	25	8.9

Table 2 shows that the mean systolic blood pressure (SBP) was the highest in the clinical department and differed significantly among departments (p<0.00001). The mean diastolic blood pressure (DBP) was also the highest in the clinical department and showed a significant difference among departments (p<0.00001). The mean pulse rate and respiratory rate did not differ significantly among clinical (78.20 ± 13.61 BPM; 16.12 ± 1.45 breaths/min), para-clinical (80.45 ± 14.70 BPM; 15.71 ± 1.46 breaths/min), and pre-clinical residents (78.10 ± 14.82 BPM; 15.70 ± 1.49 breaths/min) (p>0.05). However, clinical residents had significantly higher SBP (127.93 ± 5.15 mmHg) and DBP (84.78 ± 2.16 mmHg) than para-clinical (119.81 ± 5.18 mmHg; 73.68 ± 4.89 mmHg) and pre-clinical residents (116.90 ± 4.72 mmHg; 74.10 ± 5.15 mmHg) (p<0.00001).

**Table 2:** Comparison of vital parameters across different departments SBP: Systolic Blood Pressure, DBP: Diastolic Blood Pressure, BPM: Beats Per Minute

Characteristics	Departments	N (%)	Mean	SD	F-value	p-value
Pulse Rate (BPM)	Clinical	239 (85)	78.20	13.61	0.369	0.691
	Para-clinical	31 (11)	80.45	14.70		
	Pre-clinical	11 (4)	78.10	14.82		
Respiratory Rate (breaths/min)	Clinical	239 (85)	16.12	1.45	1.42	0.244
	Para-clinical	31 (11)	15.71	1.46		
	Pre-clinical	11 (4)	15.70	1.49		
SBP (mmHg)	Clinical	239 (85)	127.93	5.15	52.89	<0.00001
	Para-clinical	31 (11)	119.81	5.18		
	Pre-clinical	11 (4)	116.90	4.72		
DBP (mmHg)	Clinical	239 (85)	84.78	2.16	59.22	<0.00001
	Para-clinical	31 (11)	73.68	4.89		
	Pre-clinical	11 (4)	74.10	5.15		

Table 3 shows the significant differences in work patterns across departments. All clinical residents worked seven days/week, whereas all para-clinical (n=31) and pre-clinical (n=11) residents worked six days/week (χ²=28.98, p<0.0001). Emergency-duty frequency also varied significantly by department (χ²=45.12, p<0.0001), with clinical residents reporting substantially more frequent duty calls (>3 calls/week in 119 participants), while 14 of 31 para-clinical residents and all 11 pre-clinical residents had no emergency-duty calls. Sleep duration differed significantly across groups, with most clinical residents (n=129) sleeping ≤5 hours/day, whereas all pre-clinical residents slept ≥6 hours/day. These findings indicate a substantially higher workload and shorter sleep duration among clinical residents compared with the other departments.

**Table 3:** Working pattern and sleep hours across departments

Characteristics	Categories	Clinical (%)	Para-clinical (%)	Pre-clinical (%)	Total	Chi-square value	p-value
Working Days per Week	7 days	239 (100)	0	0	239	28.98	<0.0001
	6 Days	00	31 (100)	11 (100)	42		
Average Emergency/Duty Calls per Week	≤ 3 Days	120 (50)	17 (55)	00	137	5.16	<0.0001
	>3 Days	119 (50)	00	00	119		
	No emergency calls	00	14 (45)	11 (100)	25		
Average Hours Sleep per Day	≤ 5 Hours	129 (54)	00	00	129	45.12	<0.0001
	≥6 Hours	110 (46)	31 (100)	11 (100)	152		

Table 4 shows a significant difference in occupational stress across departments (χ²=62.89, p<0.00001). Clinical residents had the highest stress levels, with 119 residents in the stress category (31-45), compared to seven para-clinical and none in the pre-clinical group. Moderate stress was observed in 113 clinical, 14 para-clinical, and 6 pre-clinical residents, while no participant reported major stress. De-stressing techniques were practiced by 206 residents (179 clinical, 19 para-clinical, and 8 pre-clinical), with no significant difference among departments (χ²=2.65, p=0.267).

**Table 4:** Comparison of occupational stress scores across different departments and destressing techniques

Parameters	Categories	Clinical (%)	Para-clinical (%)	Pre-clinical (%)	Total (%)	Chi-square	p-value
Occupational Stress Score Classification	No Stress (≤ 15)	7 (3)	10 (32)	5 (45)	22 (8)	62.89	<0.00001
	Moderate Stress (16-30)	113 (47)	14 (45)	6 (55)	133 (47)		
	Stress (31-45)	119 (50)	7 (23)	0	126 (45)		
De-stressing Techniques	Yes	179 (75)	19 (61)	8 (73)	206 (73)	2.65	0.267
	No	60 (25)	12 (39)	3 (27)	75 (27)		

Table 5 shows statistically significant differences in attention, perception, and execution scores among the departments. Clinical residents required significantly more time to complete the attention, perception, and execution tests than para-clinical and pre-clinical residents (p<0.05). Although the clinical group also required a longer duration to complete the working memory test, the difference was not statistically significant (F=1.19, p=0.307).

**Table 5:** Comparison of cognitive function duration between departments All cognitive function tests are reported in milliseconds. F-value, ANOVA (analysis of variance) test statistic.

Cognitive parameters	Departments	N (%)	Mean	SD	F-value	p-value
Attention	Clinical	239 (85)	464.22	38.48	14.92	<0.00001
	Para-clinical	31 (11)	420.97	84.46		
	Pre-clinical	11 (4)	423.20	53.91		
Perception	Clinical	239 (85)	1228.85	219.44	12.35	<0.00001
	Para-clinical	31 (11)	1069.51	280.63		
	Pre-clinical	11 (4)	970.87	156.20		
Execution	Clinical	239 (85)	1435.09	203.06	3.82	0.023
	Para-clinical	31 (11)	1362.45	310.96		
	Pre-clinical	11 (4)	1276.10	221.69		
Working Memory	Clinical	239 (85)	1603.30	283.39	1.19	0.307
	Para-clinical	31 (11)	1572.65	387.12		
	Pre-clinical	11 (4)	1459.50	414.05		

Table 6 shows a significant positive correlation between occupational stress and attention (r=0.172, p=0.008), execution (r=0.249, p<0.0001), and working memory (r=0.340, p<0.0001) among clinical residents. The correlation between occupational stress and perception was positive but not statistically significant (r=0.101, p=0.120). In the para-clinical and pre-clinical groups, occupational stress showed positive but non-significant correlations with all cognitive parameters (p>0.05).

**Table 6:** Correlation between occupational stress scores and cognitive function across different departments r-value: Pearson's correlation coefficient r

Correlation	Clinical r-value (p-value)	Para-clinical r-value (p-value)	Pre-clinical r-value (p-value)
Occupational Stress Score Vs Attention	0.172 (0.008)	0.216 (0.243)	0.273 (0.455)
Occupational Stress Score Vs Perception	0.101 (0.120)	0.215 (0.245)	0.163 (0.552)
Occupational Stress Score Vs Execution	0.249 (<0.0001)	0.142 (0.445)	-0.104 (0.774)
Occupational Stress Score Vs Working Memory	0.340 (<0.0001)	0.200 (0.282)	-0.080 (0.827)

## Discussion

The present study evaluated occupational stress and cognitive functions among 281 postgraduate resident doctors from clinical (n=239), para-clinical (n=31), and pre-clinical (n=11) departments. Most participants (95.7%) were aged 25-30 years, reflecting the typical age of postgraduate trainees. Younger resident doctors often experience greater occupational stress because of the transition into demanding clinical responsibilities, heavy workloads, and adaptation to residency training, consistent with previous reports.

The study demonstrated an almost equal gender distribution among residents. Female residents exhibited significantly lower occupational stress scores than males in the clinical and pre-clinical departments, whereas no significant gender difference was observed in para-clinical departments. Cognitive performance was comparable between males and females across all departments. These findings are consistent with previous studies reporting minimal gender differences in cognitive performance, although female residents may experience additional work-life balance-related stress. Overall, gender appeared to have only a limited influence on cognitive outcomes in the present study.

A high prevalence of overweight and obesity was observed among resident doctors, which is likely attributable to irregular meal patterns, sedentary lifestyle, stress-related eating, inadequate physical activity, and disrupted daily routines during residency. Smoking, alcohol consumption, and poor exercise habits were also common, suggesting that some residents may adopt unhealthy coping mechanisms to manage occupational stress. Although many participants reported practicing stress-relieving activities, those who did not engage in such practices appeared to experience higher stress levels. These findings are in agreement with previous studies demonstrating a close association between occupational stress, unhealthy lifestyle behaviours, and increased body weight among postgraduate residents.

Clinical residents had significantly higher systolic and diastolic blood pressure than para-clinical and pre-clinical residents. In addition, a proportion of residents reported hypertension (8.9%), diabetes mellitus (4.3%), and significant sleep deprivation. These findings suggest that the cumulative effects of occupational stress, irregular work schedules, inadequate sleep, and unhealthy lifestyle practices during residency may contribute to early cardiometabolic risk. Similar observations have been reported by Sethi et al., who demonstrated that occupational stress and irregular routines increase the risk of hypertension and diabetes among postgraduate residents [13]. Similarly, Hirayama et al. identified sleep deprivation as an important contributor to occupational stress, burnout, and impaired well-being [14].

Most married residents belonged to clinical departments, whereas fewer married participants were found in para-clinical and pre-clinical specialties. Although only 9.3% of participants were married, family support may have influenced stress perception and coping ability. Previous studies have similarly suggested that unmarried residents often report greater occupational stress, whereas family support may provide psychological resilience during residency [13,14].

The majority of resident doctors experienced moderate-to-severe occupational stress, with the highest stress levels observed among clinical residents. The greater stress experienced by clinical residents is likely attributable to demanding workloads, prolonged duty hours, emergency responsibilities, sleep deprivation, higher blood pressure, and unhealthy lifestyle habits. Major stress was relatively uncommon, possibly because many residents adopted coping strategies such as relaxation techniques, hobbies, and social support. Similar findings have been reported by Hillel et al., who observed moderate-to-severe stress in approximately 70% of residents [15], while James et al. identified excessive workload and institutional demands as major determinants of occupational stress and burnout [16]. These findings suggest that individual coping mechanisms may partially mitigate the severity of occupational stress but are unlikely to eliminate its adverse effects.

Clinical residents demonstrated significantly poorer performance in attention, perception, executive function, and working memory than residents from para-clinical and pre-clinical departments. Furthermore, occupational stress was significantly associated with impaired cognitive performance among clinical residents, whereas no significant association was observed in the other departments. These findings suggest that sustained occupational stress in high-workload clinical environments may adversely affect higher cognitive functions that are essential for clinical decision-making and patient care. Potential mechanisms include prolonged activation of the hypothalamic-pituitary-adrenal axis, elevated cortisol secretion, sympathetic overactivity, sleep deprivation, fatigue, unhealthy lifestyle behaviours, smoking, alcohol consumption, and elevated blood pressure, all of which have been shown to impair attention, executive function, working memory, and decision-making. Previous studies have similarly demonstrated that occupational stress adversely affects cognitive performance among healthcare professionals [13-16].

However, these findings should be interpreted with caution. Clinical, para-clinical, and pre-clinical residents differed substantially in workload, emergency-duty frequency, sleep duration, working patterns, blood pressure, and lifestyle characteristics. These variables are themselves known to influence cognitive performance and may have contributed to the observed differences between departments. Therefore, occupational stress is likely one of several interacting factors affecting cognition during residency rather than the sole determinant. This highlights the importance of considering occupational and lifestyle-related confounders when interpreting the relationship between stress and cognitive function.

Previous intervention studies have demonstrated only modest success in reducing burnout among resident physicians. Kiratipaisarl et al. reported that individual interventions, such as coaching, mindfulness training, and resilience-building programmes, produced small but significant improvements, whereas organizational interventions alone were less effective [17]. The authors suggested that combining individual and institutional approaches may provide greater benefits. Similarly, the high prevalence of occupational stress observed in the present study indicates that institutional measures, including workload optimization, protected rest periods, improved duty scheduling, accessible mental health services, and supportive educational environments, should complement individual stress-management strategies.

A recent systematic review by Bufano et al., which included 64 studies, concluded that occupational stress, shift work, prolonged working hours, and night duties are consistently associated with impaired attention, executive function, working memory, reaction time, and decision-making among healthcare workers [18]. Resident doctors were identified as one of the groups most vulnerable to cognitive impairment because of prolonged working hours and repeated night shifts. The findings of the present study are consistent with recommendations for organizational interventions, such as reducing excessive night duties, strengthening institutional support, promoting regular rest breaks, and improving workplace environments to preserve cognitive performance and enhance physician well-being, and further emphasize the need for strategies to protect both physician health and patient safety.

The present study has several limitations. First, its cross-sectional, single-centre design precludes causal inference and limits the generalizability of the findings. Second, the unequal sample sizes, particularly the relatively small numbers of para-clinical and pre-clinical residents, reduced statistical power for subgroup analyses. Third, occupational stress was assessed using self-reported questionnaires, making reporting bias possible. Fourth, cognitive performance was evaluated at a single time point and may have been influenced by transient factors, such as fatigue, caffeine intake, sleep deprivation, anxiety, or baseline cognitive ability. Finally, differences in workload, emergency-duty frequency, sleep duration, blood pressure, and lifestyle factors across departments may have acted as confounding variables and should be considered when interpreting the observed associations.

## Conclusions

Occupational stress was highly prevalent among resident doctors, particularly among those in clinical specialties, who reported greater workload, emergency duties, and sleep deprivation. Most participants experienced moderate-to-high levels of occupational stress, while only a small proportion reported minimal stress. Higher occupational stress was associated with poorer cognitive performance, particularly in the domains of attention, perception, and task execution. Unhealthy lifestyle factors, including poor dietary habits, physical inactivity, substance use, and inadequate sleep, were also associated with higher occupational stress and less favourable health profiles. In addition, higher blood pressure was observed among residents with greater occupational stress. These findings highlight the importance of implementing institutional measures to promote stress management, healthy lifestyle practices, and mental health support for resident doctors. Such interventions may help improve resident well-being and cognitive performance, although their effectiveness should be evaluated in future studies.
